# Poor Reporting of Scientific Leadership Information in Clinical Trial Registers

**DOI:** 10.1371/journal.pone.0001610

**Published:** 2008-02-20

**Authors:** Melanie Sekeres, Jennifer L. Gold, An-Wen Chan, Joel Lexchin, David Moher, Marleen L. P. Van Laethem, James Maskalyk, Lorraine Ferris, Nathan Taback, Paula A. Rochon

**Affiliations:** 1 Kunin-Lunenfeld Applied Research Unit, Baycrest, Toronto, Ontario, Canada; 2 Ontario Medical Association, Toronto, Ontario, Canada; 3 Department of Medicine, University of Toronto, Toronto, Ontario, Canada; 4 School of Health Policy and Management, York University, Toronto, Ontario, Canada; 5 Chalmers Research Group, Children's Hospital of Eastern Ontario Research Institute, Ottawa, Ontario, Canada; 6 Toronto Rehabilitation Institute, Toronto, Ontario, Canada; 7 St. Michael's Hospital, Toronto, Ontario, Canada; 8 Clinical Epidemiology Unit, Sunnybrook Health Sciences Centre, Toronto, Ontario, Canada; 9 Centre for Research on Inner City Health, The Keenan Research Centre, Li Ka Shing Knowledge Institute, St Michael's Hospital, Toronto, Ontario, Canada; 10 Institute for Clinical Evaluative Sciences, Toronto, Ontario, Canada; 11 Department of Physiology, University of Toronto, Toronto, Ontario, Canada; 12 University Health Network, Toronto, Ontario, Canada; 13 Department of Family and Community Medicine, University of Toronto, Toronto, Ontario, Canada; 14 Department of Epidemiology and Community Medicine, University of Ottawa, Ottawa, Ontario, Canada; 15 Department of Public Health Sciences, University of Toronto, Toronto, Ontario, Canada; Copenhagen University Hospital, Denmark

## Abstract

**Background:**

In September 2004, the International Committee of Medical Journal Editors (ICMJE) issued a Statement requiring that all clinical trials be registered at inception in a public register in order to be considered for publication. The World Health Organization (WHO) and ICMJE have identified 20 items that should be provided before a trial is considered registered, including contact information. Identifying those scientifically responsible for trial conduct increases accountability. The objective is to examine the proportion of registered clinical trials providing valid scientific leadership information.

**Methodology/Principal Findings:**

We reviewed clinical trial entries listing Canadian investigators in the two largest international and public trial registers, the International Standard Randomized Controlled Trial Number (ISRCTN) register, and ClinicalTrials.gov. The main outcome measures were the proportion of clinical trials reporting valid contact information for the trials' Principal Investigator (PI)/Co-ordinating Investigator/Study Chair/Site PI, and trial e-mail contact address, stratified by funding source, recruiting status, and register. A total of 1388 entries (142 from ISRCTN and 1246 from ClinicalTrials.gov) comprised our sample. We found non-compliance with mandatory registration requirements regarding scientific leadership and trial contact information. Non-industry and partial industry funded trials were significantly more likely to identify the individual responsible for scientific leadership (OR = 259, 95% CI: 95–701) and to provide a contact e-mail address (OR = 9.6, 95% CI: 6.6–14) than were solely industry funded trials.

**Conclusions/Significance:**

Despite the requirements set by WHO and ICMJE, data on scientific leadership and contact e-mail addresses are frequently omitted from clinical trials registered in the two leading public clinical trial registers. To promote accountability and transparency in clinical trials research, public clinical trials registers should ensure adequate monitoring of trial registration to ensure completion of mandatory contact information fields identifying scientific leadership

## Introduction

Starting September 2005, the International Committee of Medical Journal Editors (ICMJE) required that all clinical trials be registered at inception in a public register in order to be considered for publication in member journals [1]. The two largest registers meeting the ICMJE standards are the International Standard Randomized Controlled Trial Number (ISRCTN) (http://www.controlled-trials.com/isrctn/) and ClinicalTrials.gov (www.clinicaltrials.gov). A major goal of trial registration is to enhance transparency and accountability in the conduct and reporting of clinical trials, an objective achieved by making details about the trial, including source of funding, methods, and design, publicly available [2], [3].

An important component of accountability in clinical research involves assigning responsibility for the conduct of trials to study ‘chairs’ or principal investigators, who assume the scientific leadership for these studies. Linking a given trial to an individual as a matter of public record advances scientific integrity by ensuring such individuals are available to address any questions or concerns that may arise from patients, their physicians, or other researchers. Without the ability to link trials to an individual, accountability remains a concern. Moreover, this information can then be compared to the investigators listed as authors, thereby providing a mechanism for ensuring that those responsible for the trial's conduct also assume public responsibility for the trial's outcome.

We reviewed a sample of clinical trial register entries in the ISRCTN and ClinicalTrials.gov to evaluate the completeness of data provided on scientific leadership and trial contact information.

## Materials and Methods

We used the metaRegister of Clinical Trials (mRCT) to simultaneously search entries in ISRCTN [5] and ClinicalTrials.gov [4], the two largest public international clinical trial registers. The ISRCTN register is owned by ISRCTN, a not-for-profit organization, and administered by Current Controlled Trials Ltd. [5]. It was formally launched in May 2003. ClinicalTrials.gov is an Internet-based register administered by the National Library of Medicine [4]. The site became active in February 2000. We used the metaRegister of Clinical Trials (mRCT) to simultaneously search entries in both registers [6].

As shown in Table 1, ClinicalTrials.gov requires that a central contact and overall study official be listed in the register entry for all trials. For the ISRCTN, the ‘priniple investigator’ must be identified. Providing his/her contact information, including name, address, and e-mail is compulsory for all trials [7]. Providing a telephone number is not compulsory. ClinicalTrials.gov mandates that the name, degree, role, and affiliation of the ‘person(s) responsible for the overall scientific leadership of the protocol, including study principal investigator’ be specified for non-IND/IDE studies [trials that are not associated with United States FDA Investigational New Drug (IND) or Investigational Device Exemption (IDE) applications] [8]. ClinicalTrials.gov also requires that the name, degree, phone number, and e-mail of a central contact be provided for the overall project.

**Table 1:** International Standard Randomized Controlled Trial Number (ISRCTN) Register and ClinicalTrials.gov adherence to World Health Organization (WHO) Minimum Registration Data Set criteria

WHO Item	ISRCTN	ClinicalTrials.gov
Unique trial number	Generated by register	Compulsory
Trial registration date	Generated by register	Compulsory
Secondary IDs	Compulsory	Optional
Funding source(s)	Compulsory	Compulsory
Primary sponsor(s)	Compulsory; includes sponsor e-mail	Compulsory
Secondary sponsors(s)	Compulsory; includes sponsor e-mail	Compulsory
Responsible contact person (Public contact person for the trial)	Compulsory; includes contact e-mail	Compulsory; includes contact e-mail
**Research contact person (Person to contact for scientific inquiries about the trial)**	**Compulsory (field is ‘Contact Name’)**	**Compulsory (field is ‘Study Chairs or Principal Investigators’)**
Title of the study	Compulsory	Compulsory
Official scientific title of the study	Compulsory	Optional
Countries of Recruitment*	Compulsory	Compulsory
Condition	Compulsory	Compulsory
Intervention(s)	Compulsory	Compulsory
Key inclusion and exclusion criteria	Compulsory	Compulsory
Study type	Compulsory	Compulsory
Anticipated start date	Compulsory	Optional
Target sample size	Compulsory	Optional
Recruitment status	Compulsory	Compulsory
Primary outcome	Compulsory	Optional
Key secondary outcomes	Compulsory	Optional

* Changed in the latest Trial Registration Data Set from ‘research ethics review’ [9]. This change has not yet been reflected in either register.

The WHO Trial Registration Data Set provides a minimum set of requirements it considers should be included in a trial register [9]. The Data Set was developed by WHO in an effort to promote global standardization in trial registration and has been endorsed by ICMJE [10]. The WHO Registration Data Set mandates the listing of a ‘Research contact person’, described as the ‘person to contact for scientific inquiries about the trial.’ [9] See Table 1 for a summary of the requirements of the WHO and the compliance with these requirements by ISRCTN, and ClinicalTrials.gov as stated on their respective websites.

### Sampling strategy

As we were interested in the sub-set of trials involving Canadian investigators, we searched mRCT using the term ‘Canada’ for all entries up to November 25, 2005. From the generated list of all trials identifying Canadian study sites or investigators (as either the study chair, co-ordinating investigator, principal investigator or site investigator), one of us (JG) manually extracted demographic information. For each trial identifying at least one Canadian investigator, data were collected on the following: trial title (public), funding source(s), recruiting status, study chair/co-ordinating investigator/principal investigator/site principal investigator names, and any contact e-mail specific to the trial. As the information was extracted from publicly available sources, no ethical approval was necessary.

#### Funding Source

We classified entries as having non-industry funding, industry funding, or partial-industry funding. Non-industry funded trials were defined as those entries listing a government agency, hospital, university, or other non-profit source as the sole funder(s). Industry funded trials were defined as those entries listing a private for-profit corporation, such as a pharmaceutical or medical devices company, as the sole funder. Partial-industry funded trials were defined as those entries listing both an industry funding source and a non-industry source.

#### Recruitment Status

Entries were classified as either ‘in progress’ (encompassing ‘ongoing’ ISRCTN entries and ‘not yet recruiting’ and ‘recruiting’ entries in ClinicalTrials.gov) or ‘no longer recruiting’ (encompassing entries listed as ‘completed’ or ‘stopped’ in ISRCTN entries and entries in ClinicalTrials.gov listed as ‘no longer recruiting’, ‘completed’, ‘suspended’, or ‘terminated’).

### Outcomes and Analyses

Our main study outcomes were the proportion of entries providing the names of those responsible for the scientific leadership of the trial (PI or Study Chair or Site PI) and the proportion of entries providing contact e-mail addresses. Among our primary outcome measures was trial e-mail contact information, a variable included among the required registration parameters for both ISRCTN and Clinicaltrials.gov, as mandated by the WHO (Table 1). While telephone contact information was initially considered as an additional measure, it is not a required field in either ISRCTN or Clinicaltrials.gov, and would not be a reliable variable across the meta-register. Also, the meta-register is an electronic medium for trial registration, necessitating on-line e-mail access for successful registration. E-mail is a widely-accepted means of communication allowing for a traceable and transferable electronic record of correspondence. Taken together, these factors support the reliability of e-mail contact information as a primary outcome measure for our analyses.

To determine whether availability of information varied according to recruitment status (in progress versus no longer recruiting), funding source (non-and partial-industry versus industry-funded), and trial register (ClinicalTrials.gov versus ISRCTN), we ran two logistic regression models with presence of scientific leadership information and presence of e-mail contact information as response variables. As we wished to examine whether trials initiated and controlled solely by industry were different from those that were not, for the logistic regression model, we grouped partial-industry and non-industry funded trials together: none of these would be as susceptible to full industry control as trials funded solely by industry. The six entries failing to list a funding source were excluded from the logistic regression model. We also used a chi-square test statistic to assess for an association between the trial register and recruitment status. Statistical analyses were performed with Stata 8 (Stata Corp, College Station, Texas) and SAS 9.1 (2002–2003, SAS Institute Inc., Cary, North Carolina).

## Results

### General

**Figure 1:**
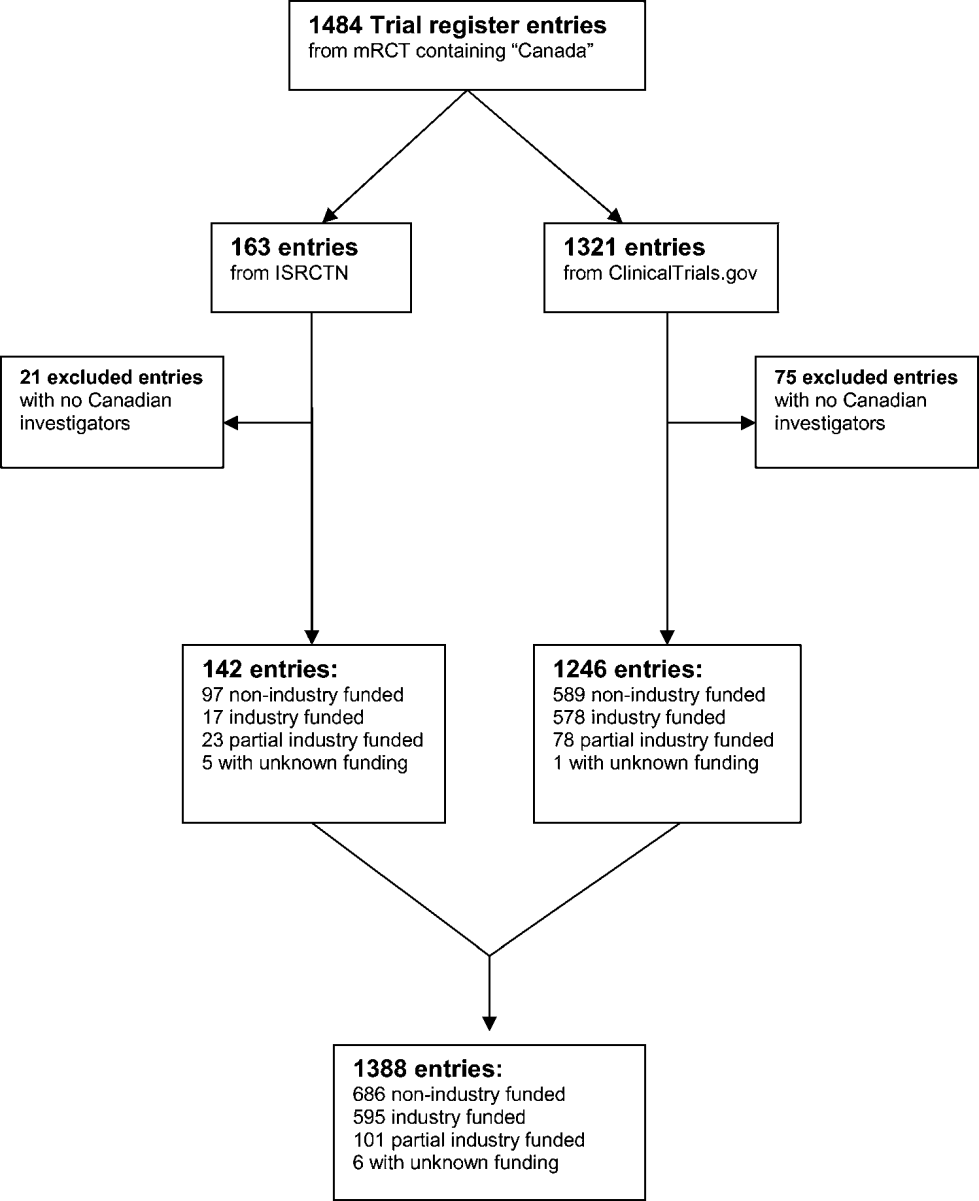


Our search yielded 1484 entries in mRCT that included the search term ‘Canada’. Of these, 163 were from ISRCTN and 1321 from ClinicalTrials.gov. Entries mentioning Canada in the abstract or in another context, but without Canadian investigators, were excluded (n = 96). Resultantly, a total of 1388 entries (142 from ISRCTN and 1246 from ClinicalTrials.gov) comprised our sample (Figure 1).

All 1388 entries provided the public study title. A total of 1382 (99.6%) listed the funding source: 686 (50%) were non-industry funded; 595 (43%) were industry funded; and 101 (7%) were partial-industry funded. Six and 36 entries respectively did not provide information about funding source or recruitment status.

### Listing of Investigator Names

Overall, 1033/1388 (74%) of trial entries listed the name of the study chair, PI, co-I, or site PI. For entries reporting recruiting status, both non- and partial-industry funded trials were significantly more likely than industry funded trials to list the name of the study chair, PI, co-I, or site PI. Of 663 non-industry funded trial entries, 659 (99%) provided this information compared to only 239 (41%) industry funded trials (Table 2). All 95 partial-industry funded trials reporting recruiting status provided information on scientific leadership. The adjusted odds ratio (OR) for non- or partial-industry funded trials providing names compared to industry trials was 259 (95% CI: 95–701).

**Table 2:** Principal investigator/study chair availability in ISRCTN and ClinicalTrials.gov by funding source and recruitment status

Recruitment status	Non-industry funded n = 663	Partial-industry funded n = 95	Industry funded n = 590	Overall n = 1348
**In progress n = 666**	372/372 (100%)	68/68 (100%)	111/226 (49%)	**554/666 (83%)**
**No longer recruiting n = 682**	287/291 (99%)	27/27 (100%)	128/364 (35%)	**442/682 (65%)**
**Total n = 1348**	**659/663 (99%)**	**95/95 (100%)**	**239/590 (41%)**	**993/1348 (74%)**

Recruitment status was also associated with the provision of scientific leadership information (Table 2). Studies classified as ‘in progress’ were significantly more likely to list this information than studies that were classified as ‘no longer recruiting’ (83% vs. 65%, OR = 1.9, 95% CI: 1.3–2.6). In examining the relationship between register and provision of scientific leadership information, since 100% of partial-industry funded studies provided names, the maximum likelihood estimate is not possible for partial-industry vs. industry. If we removed partial industry from funding source, applying a logistic regression model to compare non-industry and industry, the odds ratio was 242 (95% CI: 89–655). Non-industry funded trials were still more likely to provide names compared to industry trials.

### Listing of E-mail Contact Information

Over one third (n = 533; 38%) of trial entries provided mandatory e-mail contact information. Funding source was associated with availability of contact e-mail information for those entries reporting their recruiting status. Of 663 non-industry funded trials reporting recruiting status, 340 (51%) provided e-mail contact information compared to 88 of 590 (15%) industry funded trials (Table 3). Of 95 partial-industry funded trials reporting recruiting status, 69 (73%) provided e-mail contact information. The adjusted odds ratio for non-or partial-industry funded trials providing e-mail addresses compared to industry trials was 9.6 (95% CI: 6.6–14). When we divided funding source into three groups (non-industry, partial industry, and industry) and applied logistic regression models, the odds ratio for non-industry vs. industry was 6.0 (95% CI: 4.6–7.9), and the odds ratio for partial-industry vs. industry was 15.1 (95% CI: 9.1–25.1).

**Table 3:** E-mail contact availability in ISRCTN and ClinicalTrials.gov by funding source and recruitment status

Recruitment status	Non-industry funded n = 663	Partial-industry funded n = 95	Industry funded n = 590	Overall n = 1348
**In progress n = 666**	321/372 (86%)	63/68 (93%)	84/226 (37%)	**468/666 (70%)**
**No longer recruiting n = 682**	19/291 (7%)	6/27 (22%)	4/364 (1%)	**29/682 (4%)**
**Total n = 1348**	**340/663 (51%)**	**69/95 (73%)**	**88/590 (15%)**	**497/1348 (37%)**

We also found an association between recruitment status and availability of contact e-mail information (Table 3). Studies classified as ‘in progress’ were significantly more likely to list e-mail contact information than studies that were classified as ‘no longer recruiting’ (70% vs. 4%, OR = 260, 95% CI:118–570) (Table 3). Furthermore, we found an association between trial register and provision of e-mail addresses, with ISRCTN registered trials being more likely to provide this information (OR = 0.01, 95% CI: 0.004–0.03).

### Variation by Trial Register: Recruitment Status

We examined whether information on recruitment status was provided in each register. We determined that recruitment status was reported significantly more often by entries in ClinicalTrials.gov than in ISRCTN (99% versus 75%, χ^2^ = 305, p<0.0001).

## Discussion

Our results reveal that a substantial proportion of registered clinical trial entries are non-compliant with providing critical study e-mail contact information that is mandated by the registry. Industry funded trials were significantly less likely to both identify individuals primarily responsible for scientific leadership and to provide trial e-mail contact addresses. Studies that were no longer in the recruitment phase were also less likely to provide this information. Significant variation in compliance was found between entries listed in the two trial registers. Similar data has recently been reported elsewhere [11].

Clinical trial registration is still in its infancy. Thus, it is not surprising that in the months following the ICMJE's September 2005 deadline for mandatory registration, quality issues have become apparent. Our work demonstrates the discrepancies between non-industry and industry funded trials with respect to data entry. Our results also indicate that the data currently being entered into clinical trial registers, regardless of funding source, are often not in compliance with either the standards set forth by the registers themselves or the WHO Registration Data Set, specifically for the WHO criteria for both ‘responsible contact person’, including e-mail, and ‘research contact person’, representing scientific leadership (Table 1).

Around the time that we undertook our study, clinical trial registries were inundated with newly registered trials as a result of the introduction of mandatory registration. Instituting methods of quality assurance, such as verification of registered e-mail addresses using a unique registration number would be a valuable means of ensuring information initially entered into the trial's registration profile is valid. It is possible that similar means of quality control have been initiated since we undertook our study. This is an important issue, which should be addressed in the future. Nevertheless, it is easy to pass off responsibility for provision of this mandatory information on deficient software, or electronic limitations. However, one should not overlook that investigators or sponsors conducting clinical research are frequently using these limitations as loopholes for omitting valuable information that should allow a given study to be traced back to a responsible individual.

Our results are of importance to several stakeholder groups, including patients, health care professionals, and systematic reviewers with a vested interest in the successful development of clinical trial registers. Although considerable effort has been made to improve the quality of reporting in primary studies such as randomized controlled trials, many of these reports are published with incomplete information [12], [13]. Systematic reviewers require the ability to communicate with the study authors to seek clarifications and/or additional data, making the need to contact investigators even more critical for unpublished studies.

Mechanisms for improving compliance are necessary to ensure that trial registration continues in a manner that is consistent with the goals of WHO and ICMJE. Unless all mandatory fields are completed, the ICMJE currently does not consider a trial registered [10]. Registers could consider withholding assignment of a registration identification number for trials with incomplete data fields, or those trials listing contact information that has not been confirmed to be both valid and current. Additional adherence measures should be developed at a policy level among governments, ethics committees, and funding agencies.

Our work has one limitation. The present study is a part of a large-scale study evaluating Canadian Academic Health Sciences Centres, and thus we limited our cohort to trial entries listing at least one Canadian researcher among the contributing study investigators. As studies conducted at Canadian sites, and those involving Canadian investigators, have been demonstrated to conform to higher research standards with respect to reporting clinical data [14], and to have stricter privacy rules regulating the dissemination of personal information, our data sample may not be entirely representative of an international cohort of registered clinical trials. There are data to suggest that clinical trials conducted in other countries might have systematically different (biased) results. Randomized clinical trials (RCTs) conducted at Canadian sites are significantly less likely to report uniformly positive results than are RCTs conducted in east Asia and eastern Europe [14]. Accordingly, studies conducted at Canadian sites and/or those involving Canadian researchers may positively impact the conduct and integrity of a clinical trial. That our sample, which was limited to this very population, observed major deficiencies in proper trial registration and accountability in the clinical research indicates a significant problem even amongst clinical research supposedly conducted with a higher set of standards. Resultantly, it is likely that our sample may have under-represented the problem and provided a conservative estimate of the prevalence of non-compliance in trial registration. Additionally, identification of a responsible study contact person is amongst the WHO's minimum data set for all registered clinical trials. This is a mandatory piece of contact information for all trials conducted at any international study site, and in this respect, trials involving Canadian investigators are not systematically different than any others.

Our work is consistent with previous studies revealing problems with data quality in clinical trial registration [11], [15]. Zarin et al [15] demonstrated that in 2670 studies registered in ClinicalTrials.gov, 24% failed to enter any information in the WHO criterion ‘Primary Outcome Measure’ field. For the remaining 76%, the level of detail entered was highly variable. Another study comparing 21 different trial registers demonstrated that only 54% of entries in various registers provided adequate contact information, and less than 30% contained information on essential components of a study, such as outcome measures and intervention details [11].

### Conclusions

We found deficiencies in the availability of information related to scientific leadership and contact information in two major trial registers, with trials funded by industry and trials no longer recruiting being the least compliant. Trial registration may be a valuable mechanism for promoting transparency in clinical trials, but methods for improving compliance with the provision of registration data should be established. A failure to link trials to study chairs or principal investigators undermines the accountability and transparency that trial registration is intended to promote. Involvement in a clinical trial should be a matter of permanent public record. Transparency must not be selective in nature.
